# Supplementary material for: Determinants of left ventricular structure, filling and long axis function in systemic sclerosis
Source: PLoS One. 2021 Oct 22;16(10):e0258593. doi: 10.1371/journal.pone.0258593 (PMC8535357; doi:10.1371/journal.pone.0258593)
Supplement: Attachment — Submitted filename: Plos One SSc study Response to reviewers July 25 2021.docx [file pone.0258593.s002.docx]

This article is of potential interests and may provide novel insights into characterization of myocardial involvement in patients with systemic sclerosis, but all reviewers pointed out a number of critical issues that require amendment and improvement. Especially, lack of correction for multiple comparisons, lack of age and sex-matched healthy control group to compare, and lack of data on global longitudinal strain are critical issues of this study. We are happy to re-evaluate this article if the authors believe that they are able to address those important issues adequately in the revised version.

Please submit your revised manuscript by May 23 2021 11:59PM. If you will need more time than this to complete your revisions, please reply to this message or contact the journal office at [plosone@plos.org](mailto:plosone@plos.org). When you're ready to submit your revision, log on to <https://www.editorialmanager.com/pone/> and select the 'Submissions Needing Revision' folder to locate your manuscript file.

Please include the following items when submitting your revised manuscript:

- A rebuttal letter that responds to each point raised by the academic editor and reviewer(s). You should upload this letter as a separate file labeled 'Response to Reviewers'.
- A marked-up copy of your manuscript that highlights changes made to the original version. You should upload this as a separate file labeled 'Revised Manuscript with Track Changes'.
- An unmarked version of your revised paper without tracked changes. You should upload this as a separate file labeled 'Manuscript'.

If you would like to make changes to your financial disclosure, please include your updated statement in your cover letter. Guidelines for resubmitting your figure files are available below the reviewer comments at the end of this letter.

If applicable, we recommend that you deposit your laboratory protocols in [protocols.io](http://protocols.io/) to enhance the reproducibility of your results. Protocols.io assigns your protocol its own identifier (DOI) so that it can be cited independently in the future. For instructions see: <http://journals.plos.org/plosone/s/submission-guidelines#loc-laboratory-protocols>. Additionally, PLOS ONE offers an option for publishing peer-reviewed Lab Protocol articles, which describe protocols hosted on [protocols.io](http://protocols.io/). Read more information on sharing protocols at <https://plos.org/protocols?utm_medium=editorial-email&utm_source=authorletters&utm_campaign=protocols>.

We look forward to receiving your revised manuscript.

Kind regards,

Masataka Kuwana, MD, PhD
Academic Editor
PLOS ONE

Journal Requirements:

When submitting your revision, we need you to address these additional requirements.

1. Please ensure that your manuscript meets PLOS ONE's style requirements, including those for file naming. The PLOS ONE style templates can be found at

<https://journals.plos.org/plosone/s/file?id=wjVg/PLOSOne_formatting_sample_main_body.pdf> and

https://journals.plos.org/plosone/s/file?id=ba62/PLOSOne_formatting_sample_title_authors_affiliations.pdf

2. Please include captions for your Supporting Information files at the end of your manuscript, and update any in-text citations to match accordingly. Please see our Supporting Information guidelines for more information: http://journals.plos.org/plosone/s/supporting-information.

[Note: HTML markup is below. Please do not edit.]

Reviewers' comments:

Reviewer's Responses to Questions

**Comments to the Author**

1. Is the manuscript technically sound, and do the data support the conclusions?

The manuscript must describe a technically sound piece of scientific research with data that supports the conclusions. Experiments must have been conducted rigorously, with appropriate controls, replication, and sample sizes. The conclusions must be drawn appropriately based on the data presented.

Reviewer #1: Yes

Reviewer #2: Partly

Reviewer #3: Yes

2. Has the statistical analysis been performed appropriately and rigorously?

Reviewer #1: Yes

Reviewer #2: Yes

Reviewer #3: Yes

3. Have the authors made all data underlying the findings in their manuscript fully available?

The [PLOS Data policy](http://www.plosone.org/static/policies.action#sharing) requires authors to make all data underlying the findings described in their manuscript fully available without restriction, with rare exception (please refer to the Data Availability Statement in the manuscript PDF file). The data should be provided as part of the manuscript or its supporting information, or deposited to a public repository. For example, in addition to summary statistics, the data points behind means, medians and variance measures should be available. If there are restrictions on publicly sharing data—e.g. participant privacy or use of data from a third party—those must be specified.

Reviewer #1: Yes

Reviewer #2: Yes

Reviewer #3: Yes

4. Is the manuscript presented in an intelligible fashion and written in standard English?

PLOS ONE does not copyedit accepted manuscripts, so the language in submitted articles must be clear, correct, and unambiguous. Any typographical or grammatical errors should be corrected at revision, so please note any specific errors here.

Reviewer #1: Yes

Reviewer #2: Yes

Reviewer #3: Yes

5. Review Comments to the Author

Please use the space provided to explain your answers to the questions above. You may also include additional comments for the author, including concerns about dual publication, research ethics, or publication ethics. (Please upload your review as an attachment if it exceeds 20,000 characters)

Reviewer #1: I am happy to have an opportunity to review an interesting manuscript written by Dr. Peverill, et al. regarding left ventricular abnormalities in systemic sclerosis. In my opinion, there are several problems that the authors should consider to revise.

1. The aim of this study was to explain why the previous relevant studies concluded differently. The authors stated in the conclusion that multivariate analyses adjusting for the potential confounding variables could resolve the previous discrepant findings, but it sounds strange. Conclusion should be novelty. Multivariate analysis is not likely to be the novelty of this study.

#Authors response. The aim of our study was to identify variables which might have confounded the comparison of SSc and control subjects in previous studies in SSc, and thus provide possible explanations for previous divergent findings with regard to LV M-mode measures, LV filling variables, s` and e`. A single study was never going to be able to resolve all previous discrepant findings. Our main conclusion was that there are a number of possible factors which could have varied between the studies and between the SSc subjects and controls which could have influenced the results of these studies. The importance of multivariate analyses to identify such factors was shown in the current study and an important implication of this is that multivariate analysis could be used in reanalysis of previous studies to adjust for the possible contributions of confounding factors. The statement about the potential value of multivariate analysis to adjust for the identified variables in the abstract has been modified to improve clarity. The identification of the variables is the new finding of this study, a potential utility of these findings is adjustment for their potential contribution to differences between SSc and control subjects using multivariate analysis.

2. Please consider to reorder the tables. Table 1 and Table 2 are literature reviews. Since this is an original article, these tables should be behind other tables and only stated in the discussion part.

#Authors response. The Tables have been moved.

3. The number of the enrolled patients is missing in Table 3.

#Authors response.

This Table has now been renumbered as Table 1. The number of subjects is now included in the title of the table.

4. The definition of the abbreviations is missing in Table 4.

#Authors response.

Table 4 is now renumbered as Table 2. All the abbreviations are included at the beginning of the manuscript and our understanding is that this is acceptable to the editorial policy of PLOS One.

Reviewer #2: The present paper aims to solve current inconsistencies in potential associations between LV systolic and diastolic echo parameters and systemic sclerosis. Unfortunately, it falls short on several important issues, which should be discussed:
- The authors tested a plethora of different associations, without any attempt for multiple comparison correction. Having such a high number of potential correlations, it is natural that some of them could reach statistical significance by chance. Therefore, the proposed associations (LVEDD and BSA, SWT and age, LVEDD and diffuse disease, age and A, DT, and E/A, HR and E/A), which the authors advocate as useful in solving the current "dilemma", could have been produced by chance. A multiple comparison correction should be implemented for the whole statistical analysis to strengthen the results, otherwise, the discussion is not supported by the data and only tries to make sense of the hundreds of statistical tests performed.

#Authors response. We agree that testing for multiple associations has the potential for false positive findings, particularly if a scatter gun approach to variable selection is used. However, there are some important distinctions to be made about this study with respect to the suggestion that we should have made adjustments for multiple comparisons. First, the main aim of this study was to identify possible factors which could have confounded the analysis of previous studies in SSc. Adjusting for multiple comparisons has less relevance in this situation. In other words, there are reasons to slightly favour the possibility of false positives compared to the possibility of false negatives in this circumstance. Second, the variables tested were selected based on previous reports in healthy subjects, previous reports in SSc subjects, or on a limited amount of hypotheses arising from plausible associations with SSc-disease specific features. Therefore, examples such as positive correlations of BSA with LVEDD, age with SWT, age with A, E/A and DT, and heart rate with E/A and DT could not be assumed in SSc, but were expected based on extensive data in healthy subjects, and thus are not likely to be due to chance.

- Regarding the vast majority of statistical associations tested who did not reach statistical significance, the authors should take into consideration that the sample size is very low (n=100) and therefore not reaching the p-value can be inconclusive. Nonetheless, throughout the text and the discussion, all p values > 0.05 were considered as lack of association, whereas the low statistical power should explain many of the results.

#Authors response. We agree that a p value >0.05 does not mean that there is no association, only that there is no evidence for an association. We make no claims that we have proved that there are variables with no connection to LV structural and functional variables in SSc. However, a p value >0.05 in a regression analysis of 100 subjects does suggest that any genuine association missed due to insufficient numbers is unlikely to be a close one.

- Why was global longitudinal strain not considered? It is nowadays the best parameter for detecting subclinical progression of systemic sclerosis-related cardiomyopathy and can be retrieved offline. Moreover, it could have made the paper less obsolete.

#Authors response. We agree that GLS is of interest in SSc but certainly do not agree that GLS makes all other LV variables obsolete. For example, GLS is a marker of long-axis contraction and thus provides no direct information about LV short axis diameter, wall thickness or diastolic function. Furthermore, even if GLS is abnormal in SSc it would still be important to understand the associations of GLS, e.g. s` and GLS would be expected to be correlated. Most pertinent for the current study, however, GLS was not included because it is important, this study was already long, and a study of GLS would require a substantial increase in the methodology, results and discussion in an already long manuscript.

- There are many large studies that details echo parameters that were not included in Table 2 (Eur J Prev Cardiol 2018; 25: 1598-1606; Eur Heart J Cardiovasc Imag 2012; 13: 863–870; Cardiovasc Ultrasound 2014; 12: 13 and probably many others). Therefore, the table is not complete and, therefore, not informative.

#Authors response. We agree that the list of studies is not complete. However, this was not a review paper and the aim of the Tables were to show that there are a substantial number of discrepancies between previous reports in SSc, and also to highlight that potentially relevant patient demographic data are missing from a number of previous studies. It would certainly be important if there was a study investigating the effects on the left ventricle of SSc that we had not mentioned, which had provided all the relevant information, and also adjusted for all potential confounding variables, but we are unaware of such a study. The first mention of the relevant tables (now renumbered 6 and 7) is now in the first paragraph of the discussion and it now makes the point that the tables include a selection of relevant studies.

- One of the potential limitation of the present paper is the inclusion of patients with a history of disease from 0 to 40 years. This is a very heterogeneous population, and the effect over time of systemic sclerosis-related cardiomyopathy could not be linear (Eur J Prev Cardiol 2020; 27: 1876–1886), therefore making the presented results more difficult to interpret.

#Authors response. We agree that there is heterogeneity in the study group. This heterogeneity has both benefits and hazards. If the aim is to determine whether there are correlations with disease duration, then variation in disease duration is necessary. However, we also agree that the effect of SSc over time may well not be linear. Age was correlated with log disease duration in the current study so it is important to consider this relationship when evaluating the potential effects of disease duration. A limitation of some previous studies which have investigated the effects of disease duration is that they have not adjusted for age. We would also point out that the limitations of the current study regarding assessment of the effects of disease duration are already mentioned in the discussion.

Reviewer #3: Determinants of left ventricular structure, filling, and long-axis function in systemic
sclerosis
A cross-sectional study evaluating the possible effect of disease type, DD, the presence of ILD, age, body size, sex, BP, heart rate, and history of hypertension on the LV structure, transmitral flow, and LV long-axis function.
Comments:
The main weakness of the study is the lack of age and sex-matched healthy control group to compare and investigate the relationship between the above-mentioned variables and left LV structure, transmitral flow, and LV long-axis function. The authors used the cut-off values for the determinants defined by 2016 ASE/EACI criteria, however, it would be better to include a control group concurrently to compare the results outside the normal limits.

#Authors response

The aim of this study was to identify variables which could have confounded previous echocardiographic studies in SSc given the inconsistency of previous LV findings. Our aim was not to compare SSc and control subjects. A study in which there was another comparison of SSc and control subjects would certainly be of value, but would not necessarily resolve all these discrepancies, particularly in view of the findings of the present study with respect to the possible confounding factors. Furthermore, a manuscript which addressed the aim of our study and also included a control group would be of unmanageable size. On the other hand, the identification of possible confounding factors provides options for re-analysis of previous studies with adjustment for these factors and for planning future studies which include control groups.

1. Introduction:
In the introduction, the targetted subject of the study is explained in a very scattered way. By keeping the tables (Table 1 and 2) for discussion and giving a summary of the past studies, you might write a more impressive and summary introduction. I would kindly recommend you give a clear and concise insight into the topic for the introduction.
Language editing is recommended for the Introduction.

#Authors response. Thank you for this suggestion. We have made a number of changes in an attempt to make the introduction more concise and aid in its clarity.

2. Methods:
In the first paragraph of the section “Subjects", you state that you enrolled the patients fulfilling the three different classification criteria. You should better use only one classification criteria, preferably the 2013 ACR/EULAR classification criteria. And for classifying the patients into limited and diffuse subtypes, it is better to use the LeRoy 1988* criteria.
* LEROY EC, BLACK C, FLEISCHMAJER R, et al.: Scleroderma (systemic sclerosis): classification, subsets, and pathogenesis. J Rheumatol 1988;15(2):202–205

#Authors response.

The references have been changed as suggested.

3. Results
In Table 6, there are the results of multivariate regression analysis of two dependent variables (LVEDD and SWT). However, in the text above the table, you report the results about PWT and RWT. I suppose you included the variables with meaningful results in the table. However, you should mention this in the text or otherwise show all the results in Table 6. I have the same comments about Table 7.

#Authors response.

These comments now refer to the renumbered Table 4 and Table 5. We have slightly changed the titles of these 2 tables to reflect that they are selected models. There were already statements in the text regarding the choice of the dependent variables. PWT was not included separately because the findings were similar to SWT. RWT was not included in the table because it was only correlated with age. Only independent variables with more than one dependent correlate were included in Table 5. The statement in the text has been rewritten to improve its clarity.

4. Discussion
You should better emphasize the main and most important results of the study in the first paragraph of the Discussion. Considering the large amount of data analyzed in this study, it seems difficult to shorten the discussion. However, I would kindly recommend you to review the manuscript in terms of a brief and strong discussion by avoiding the unnecessary repetition of the results,

#Authors response. Thank you for these suggestions. A number of changes to the discussion have been made.
