# Supplementary material for: Determinants of left ventricular structure, filling and long axis function in systemic sclerosis
Source: PLoS One. 2021 Oct 22;16(10):e0258593. doi: 10.1371/journal.pone.0258593 (PMC8535357; doi:10.1371/journal.pone.0258593)
Supplement: Attachment — Submitted filename: Responses to Reviewer comments 2.docx [file pone.0258593.s003.docx]

Responses to Reviewer 3 comments

\# It should be noted that in a disease such as systemic sclerosis, where clinical manifestations and disease activation are not always correlated with disease duration, confounding factors cannot be compared as easily as in the normal population. The authors should then have considered including some disease-specific variables (such as the modified Rodnan skin score or EUSTAR disease activity index) in their multivariate analysis.

\# Author’s response. We agree that clinical manifestations and disease activation may not be correlated with disease duration in systemic sclerosis (SSc), and also that there could well be factors which affect the left ventricle which are different to the normal population. The study does already include SSc disease-specific variables in the analysis other than disease duration, specifically disease type and interstitial lung disease. Furthermore, we already discuss the limitations of disease duration as an independent variable in the manuscript. Nevertheless, disease duration is a variable which has been reported to be a determinant of LV variables in previous studies and we believe that further testing of its association with LV variables in SSc was justified. Our study is certainly not the last word on the left ventricle in SSc, and disease-specific variables other than the ones we have studied and reported on could turn out to shed further light on the topic. However, the information requested regarding the Rodnan skin score and EUSTAR disease activity index is not available in all our subjects. Moreover, we fail to see how the absence of these variables invalidates either the originality, importance or the validity of our findings as currently presented.

\# Since systemic sclerosis is a rare and special disease, making inferences as in studies in the normal population may result from not knowing the disease well enough.

#Author’s response. We are not sure which inferences are being referred to by the reviewer, or which aspect of SSc the reviewer believes has not been understood by the authors. Moreover, we find this statement particularly surprising given that the main findings of the current study relate to a number of the same factors which affect left ventricular structural and functional variables in the normal population (e.g. age, body size, heart rate, blood pressure), and that we did find to have similar relationships with left ventricular variables in SSc. On the other hand, that we did not assume that this would be the case in SSc was part of the rationale for performing this study.

\# By agreeing with the second reviewer, I think that some relationships can come out by chance without doing multiple comparison analysis.

#Author’s response. We presume this statement relates to the issue of whether adjustments should have been made because multiple variables were tested in regression analyses. We did already address this question in a detailed response in our previous rebuttal but have restated and expanded this response below.

We agree that testing for multiple associations has the potential for false positive findings, particularly if a scatter gun approach to variable selection is used. However, the appropriateness of such adjustment depends on the aims and design of the particular study, and we believe that there are reasons why it is not necessary for the present study. First, the main aim of this study was to identify possible factors which could have confounded the analysis of previous studies in SSc, and we argue that adjusting for multiple comparisons has less relevance in this situation. In other words, there are reasons to favour the possibility of false positives compared to the possibility of false negatives in this circumstance. Second, there was no scatter gun approach to the choice of variables (i.e. a limited amount of independent variables were tested based on previous evidence). There are therefore also Bayesian issues given the considerable evidence for associations of the variables of age, sex, heart rate, blood pressure and body surface area with a number of left ventricular variables in the healthy population. In other words, our observations in SSc of correlations of BSA with LVEDD, age with SWT, age with A, E/A and DT, and heart rate with E/A and DT could not be assumed in SSc, but nevertheless were able to be postulated based on extensive data in healthy subjects, and thus are not likely to be due to chance. The SSc disease-specific variables were also carefully selected based on previous reports of their effects on left ventricular variables in SSc subjects (disease duration and interstitial lung disease) or because there was a plausible association with SSc disease-specific features (SSc type).

A possible explanation for any false positive finding in comparisons of 2 groups is that it has occurred due to unrecognized differences in one or more other variables between two groups which have had a direct effect on the dependent variable in question. It is thus worthwhile pointing out again that identifying such possible variables was the main aim of the present study. We also wish to emphasise that the positive associations reported in the current study were based on multivariable analyses which therefore provided adjustments for other contributing variables, and that the findings of such analyses are therefore more robust than positive associations that might have been present only in single variable analyses (see Slinker et al Circulation 2008 Multiple linear regression: accounting for multiple simultaneous determinants of a continuous dependent variable. V117; P1732).

\# In my view, the authors' effort to show that the results of previous SSc studies were inadequate or inaccurate is more prominent than the purpose of reporting the results of their own study in an objective way. This turns into an effort to make the readers accept the accuracy of their own results rather than putting the originality of the article forward.

\# Author’s response. It was not our intention to attempt to show that the results of previous studies were inadequate or inaccurate and we made no such claims in the manuscript. On the other hand, that there are numerous discrepancies between the findings about the left ventricle in the past SSc literature, as demonstrated in the tables, necessarily implies that some of the reported findings (negative or positive) are incorrect. This then was the rationale for our attempt to identify variables which may have confounded previous analyses. We wish to point out that this was our main study conclusion “In SSc there are associations of sex, body size, age and disease type with LV structural variables, of age and heart rate with E/A, and of age with both systolic and early diastolic LV long-axis velocities. Appropriate adjustment for these variables could help to resolve current uncertainties regarding SSc effects on the left ventricle.” We did not, and indeed could not, claim or prove the inaccuracy or inadequacy of any particular study. They findings do, however, provide a potential basis for performing appropriate adjustments in the analyses of future studies, and also in the reanalysis of previous data, with the possibilities that this could change previous false negative findings into true positives or previous false positive findings into true negatives.

We found it difficult to understand the reasoning behind the statement “This turns into an effort to make the readers accept the accuracy of their own results rather than putting the originality of the article forward”. We clearly believe in the accuracy of the findings of our study and that the results have been presented in an objective way. We are not aware of any other study which addresses the aims of the current study so we believe the originality of the study speaks for itself.
